# Desperately seeking reductions in health inequalities: perspectives of UK researchers on past, present and future directions in health inequalities research

**DOI:** 10.1111/1467-9566.12374

**Published:** 2015-11-20

**Authors:** Kayleigh Garthwaite, Katherine E. Smith, Clare Bambra, Jamie Pearce

**Affiliations:** ^1^ Department of GeographyWolfson Research Institute for Health and WellbeingDurham University; ^2^ Global Public Health UnitSchool of Social & Political ScienceUniversity of Edinburgh, Edinburgh; ^3^ Centre for Research on Environment, Society & HealthSchool of GeoSciencesUniversity of EdinburghEdinburgh

**Keywords:** health inequalities, interventions, UK, evidence‐based policy, research, advocacy

## Abstract

Following government commitments to reducing health inequalities from 1997 onwards, the UK has been recognised as a global leader in health inequalities research and policy. Yet health inequalities have continued to widen by most measures, prompting calls for new research agendas and advocacy to facilitate greater public support for the upstream policies that evidence suggests are required. However, there is currently no agreement as to what new research might involve or precisely what public health egalitarians ought to be advocating. This article presents an analysis of discussions among 52 researchers to consider the feasibility that research‐informed advocacy around particular solutions to health inequalities may emerge in the UK. The data indicate there is a consensus that more should be been done to learn from post‐1997 efforts to reduce health inequalities, and an obvious desire to provide clearer policy guidance in future. However, discussions as to where researchers should now focus their efforts and with whom researchers ought to be engaging reveal three distinct ways of approaching health inequalities, each of which has its own epistemological foundations. Such differences imply that a consensus on reducing health inequalities is unlikely to materialise. Instead, progress seems most likely if all three approaches are simultaneously enabled.

## Introduction

The UK has been recognised as a global leader in health inequalities research and policy, with recent government‐led policy efforts to reduce health inequalities being described as ‘historically and internationally unique’ (Mackenbach 2011: 1249). Yet, despite the raft of policies intended to reduce health inequalities, introduced between 1997 and 2010, the UK's health inequalities have continued to widen by most (though not all) measures – see (Bambra 2012, Barr *et al*. 2012, Thomas *et al*. 2010, Thorlby and Maybin 2010). This failure has prompted multiple commentaries and calls for advocacy to facilitate greater public support for the kinds of upstream policies that available evidence suggests may be required (Bambra *et al*. 2011, Mackenbach 2011).

In Chapman's (2007) terms, public health advocacy includes (amongst other things) working to place and maintain issues on public and political agendas (and exploiting opportunities to do so), discrediting opponents of public health objectives and working to frame evidence in persuasive ways (e.g., by using metaphors or analogies). In other words, advocacy involves strategically ‘selling’ public health objectives to a range of non‐academic audiences. This way of thinking about advocacy, which Carlisle (2000) has termed representational, implies that health inequalities researchers would first need to achieve some kind of consensus on the policy (or societal) changes they are trying to sell. Yet there is currently little agreement as to what exactly it is that public health egalitarians ought to be advocating (Horton 2012). Other definitions of advocacy are more akin to Burawoy's (2005) notion of public sociology, in which researchers engage in dialogue with members of the public, work collaboratively with organisations representing public interests and generally try ‘to make visible the invisible’ (Burawoy 2005: 264). Here, advocacy involves working with relevant communities to ensure that voices that might traditionally be ignored are given due regard; a ‘facilitational’ form of advocacy, in Carlisle's (2000) terms. In either case, advocacy clearly involves something more than the widely accepted model of researchers working with senior civil servants to try to develop evidence‐informed policy responses to health inequalities (see, for example, Petticrew *et al*. 2004).

Calls for more advocacy to reduce health inequalities therefore raise important questions for researchers about the ways in which we work to effect change. As Scambler (2012) points out, the phrase ‘tackling health inequalities’ means different things to different people:

Articulated as Weberian ideal types, for those coming from a ‘policy sociology’ perspective engagement for change involves working with people of influence; while for those coming from a ‘critical sociology’ perspective it can involve working against them (Burawoy 2005).

(Scambler 2012: 139)

This article applies a sociological lens to exploring these different ways of approaching health inequalities, reflecting Burawoy's suggestion that we should ‘apply sociology to ourselves’, in order that we become more conscious of the forces driving our research (Burawoy 2005: 285). It presents an analysis of the perspectives of 52 UK‐based researchers involved in health inequalities debates to explore how these researchers feel about the development of the field so far and what kinds of activities they think researchers should be undertaking. After briefly describing the methods and data sources, the article is divided into four sections. Firstly, we consider what the data suggest researchers feel they have learned (and not learned) from health inequalities research and policy experiences in the UK to date. Secondly, we identify areas of future research activity that the health inequalities researchers who participated in this project felt ought to be prioritized. Thirdly, we examine the kinds of activities our participants believed they ought to be undertaking in order to promote evidence‐informed change. The fourth, concluding section draws the findings together, arguing that the data identify some important divisions amongst health inequalities researchers over the kinds of work it is appropriate and desirable for researchers to undertake; schisms which appear to map onto epistemological, ontological and ideological differences. These differences require attention because they appear to be contributing to professional contestations, especially on research funding, and therefore seem likely to undermine efforts to achieve the kind of clear policy messages that public health advocacy requires. Indeed, the contrasting ways of thinking about health inequalities (and the wider world) lead not only to differing preferences for the future direction of health inequalities research but also to different understandings of the role that research, and researchers, play (and ought to play) in public policy debates.

The article builds directly on two earlier (linked) articles that explored the views of a small number of senior researchers and civil servants on evidence for tackling health inequalities (Petticrew *et al*. 2004, Whitehead *et al*. 2004). Like this earlier study, we have produced a companion article that compares the perspectives of policy actors to those of the researchers discussed in this article (Smith *et al.,* under review). As well as providing a contemporary update, our research considers the views of a broader range of researchers and policy actors representing a variety of academic disciplines, career stages and academic or policy‐related institutions (52 researchers and 58 policy actors compared with the nine senior researchers and seven senior civil servants involved in the earlier studies). Looking back on the findings of these earlier studies, the article focusing on the views of senior researchers concluded that there was, at that time, ‘significant potential for rapid progress to be made in developing both evidence based policy and policy relevant evidence to tackle health inequalities’ (Whitehead *et al*. 2004: 817). In light of subsequent claims that policy efforts to reduce health inequalities have been relatively unsuccessful (Mackenbach 2011), it seems appropriate to create space to once again reflect on the kinds of work that health inequalities researchers produce and engage in, and to consider progress and potential future directions. The planned changes to the UK's constitutional arrangements, including the devolution of key responsibilities from Westminster to some large urban local authorities in England and the governments in Scotland, Wales and Northern Ireland, as well as current fears over the likely impact of ‘austerity’‐led reforms on health inequalities, underline the timeliness of this endeavour (Bambra *et al*. 2015, Pearce 2013, Reeves *et al*. 2013).

## Methods

A 2‐day symposium was held in Edinburgh in December 2012 at which 87 participants from a range of sectors discussed the legacy of health inequalities research and policy and considered potential directions for future research. Participants were invited to the event on the basis of their involvement in health inequalities research, policy, practice or advocacy. Selection was informed by two previous qualitative research projects (Smith 2013a) and by the professional networks and expertise of a steering group (see acknowledgements). Efforts were made to ensure that researchers came from a range of disciplinary backgrounds, career stages, institutional locations and specific areas of expertise within health inequalities research.

A total of 14 one‐hour focus groups were undertaken during this event, in which 76 symposium attendants participated. There are many definitions of focus groups; we adopted a style based upon a loosely facilitated approach to generating organised discussion, as suggested by Kitzinger (1994). For the first seven (morning) sessions, participants were divided according to their profession (Table 1). Academic researchers were allocated to three separate groups, according to their primary methodological expertise (which was ascertained via their website profiles and checked via e‐mails prior to the event): (i) quantitative, (ii) mixed methods and (iii) qualitative. This division was made to ensure that the focus groups provided a range of views from researchers working in different academic traditions and with a variety of epistemological perspectives. Researchers working in public sector and policy settings were allocated to other groups. In the context of this article, the term researcher includes individuals involved in research working across a range of settings (e.g., in knowledge broker organisations and the public sector, as well as academia) and applied to 52 symposium attendants (see Tables 1 and 2). All these individuals had published articles in academic journals focusing on health inequalities or the social determinants of health, and health inequalities in the UK were a primary research focus for most. However, a small number of researchers were invited to participate because they had a relevant area of expertise that would otherwise not be represented (this included two health economists and two political scientists focusing on corporate policy influence, all of whom had an interest in health equity). The disciplinary backgrounds of participants varied widely and included anthropology, economics, epidemiology, geography, public health medicine, political science, social policy and sociology.

**Table 1:** Focus group participants (*N *=* *76)

Participants in the first focus small group discussions	
Focus group 1	11 researchers who primarily employ quantitative methods
Focus group 2	10 researchers who primarily employ mixed methods
Focus group 3	10 researchers who primarily employ qualitative methods
Focus group 4	11 (2 of whom were researchers), who primarily identified themselves as being involved in public health advocacy
Focus group 5	14 individuals (of whom 4 were researchers) working in public health policy and practice
Focus group 6	11 individuals (of whom 8 in one group were researchers) involved in public health knowledge exchange
Focus group 7	9 individuals (of whom 7 were researchers) in another group were involved in public health knowledge exchange
	Total: 52 involved in research

**Table 2:** Participants in the second focus group discussions (*N *=* *70)

Total no.	Involved in research	Methodology of those involved in research	Chosen topic
7	4	4 academics (all quantitative/mixed methods)	Research agendas beyond health and what can we do to reduce health inequalities?
8	7	5 qualitative academics, 1 knowledge broker (with a quantitative background) and 1 individual working on research in a policy setting (with mixed methods experience)	Lived experiences of health inequalities
10	8	4 quantitative academics, 1 qualitative academic, 1 individual working with research in a local policy setting, 1 researcher working in national policy contexts and 1 researcher working in a knowledge broker organisation.	Participatory research and policy
10	7	4 mixed methods or quantitative academic researchers and 3 public sector researchers.	Evaluation
7	5	4 quantitative academic researchers and 1 public sector researcher.	Evaluation
14	6	3 primarily employ mixed methods, 1 quantitative and 2 qualitative	Welfare reform/retrenchment
14	11	2 qualitative academics, 1 mixed methods academic, 2 quantitative academics and 6 researchers working in public sector/policy settings	Encouraging researchers, policymakers and practitioners to work collaboratively
Total	48		

During the first set of focus group discussions, participants were asked to generate a list of suggestions for future health inequalities research agendas. Participants were then asked to indicate which topics they were most interested in discussing further and were allocated to one of seven afternoon focus group discussions on this basis (see Table 2). A small number of participants were unable to stay for the whole day (so 52 researchers participated in the first set of focus groups and only 48 in the second set). Each focus group was facilitated by a member of the steering group and participants were asked to discuss the questions outlined in Box 1.

Box 1 **Main questions for participants in the focus group sessions**

Questions for the first seven focus groups:

1. What kinds of research should researchers working to reduce health inequalities focus on?
2. What should health inequalities researchers be doing (if anything), beyond academic work, to support efforts to reduce health inequalities?

Questions for the second seven focus groups:

1. What specifically would this new research agenda involve?
2. What needs to happen to facilitate/enable this kind of research activity?
3. What, if any, are the potential problems with developing this kind of research agenda?

Discussions took place under the Chatham House rule, which enabled all participants to share the content of discussions with others but only on a non‐attributable basis. All participants were asked to sign written consent forms enabling the focus groups to be digitally recorded and transcribed, before being anonymised (by KS). Two authors (KG and KS) read all 14 transcripts and jointly developed a thematic coding framework. The transcripts were then coded using NVivo 10 software (KG and KS each led on coding half the transcripts and then cross‐checked the other seven transcripts for consistency). The anonymised, coded transcripts were made available to the other steering group members for further analysis.

## Results

### What have we learned so far? Researchers' reflections on the legacy of health inequalities research and policy in the UK

#### Post‐1997 policy advice and limited learning

The findings reveal there is widespread concern among researchers about the failure to adequately learn from the multiple policies and interventions intended to tackle health inequalities in the post‐1997 UK context. Participants attributed this failure to the complexity of health inequalities and the policies intended to address these inequalities as well as to research funding and methodological constraints.

Several academic researchers specifically picked up on Mackenbach's (2011) article, which had been circulated in advance and which argues that, despite some partial successes, the English strategy failed to reach its own targets of a 10 per cent reduction in inequalities in life expectancy and infant mortality. This claim was disputed by a small number of researchers, particularly with regard to health inequalities between Spearhead and other areas. The Spearhead group of 70 local authorities and 62 primary care trusts was introduced in 2004 in the 88 most health‐deprived areas in England as the focus of government interventions designed to reduce health inequalities (Department of Health 2004). More recent analysis seems to support Mackenbach's (2011) assessment. Barr *et al*. (2012), for example, present data which suggest that, despite some success, overall people in Spearhead areas did not experience the improvement in health that was promised. Indeed, one of the main areas of agreement among the researchers participating in the symposium seemed to be that efforts to tackle health inequalities in the UK had been less successful than people had hoped, and this was perceived to be at least partly because health improvements had been greater for those already doing well.

Beyond this broad assessment, there was a palpable frustration amongst many researchers that national‐level analyses have ignored the potential for learning lessons about what has (and has not) worked amongst the multitude of different policies and interventions that were implemented across the UK between 1997–2012:

I think we really need to pin down what's worked in the last 15 years. And when I re‐read the Mackenbach paper … I felt a little bit annoyed … . It must have been relatively easy … to go back and pull out some key policy documents and then look at the targets. And you hear them say, ‘okay, well folks this didn't work’. But … I think we need to be really clear … about what we've learned over the past 15 years, and what we're confident about and what we don't know about. (Academic)

As several participants noted, a failure to reduce health inequalities at a national level does not rule out the possibility that some interventions and policies were successful but that the impacts were countered by other policies, or that health inequalities policies prevented the gradient from getting even steeper. Responding to suggestions made by some policy actors at the event that there are examples of geographical areas in the UK in which health inequalities have been reduced, researchers widely agreed that not enough effort has gone into collating the multiple analyses and evaluations that have been undertaken of local and national policies and interventions in the UK. There was concern that, without this kind of detailed, comprehensive analysis, it would remain difficult for health inequalities researchers to adequately support future decision‐making.

#### A lack of clear policy solutions

In addition, some researchers expressed frustration at what they perceived to be an ongoing reticence within the research community to provide clear policy guidance as to the kinds of policies most likely to reduce health inequalities:

Academic I'm remembering … when the ‘97 Labour government came in and said they wanted to do something about health inequalities, they looked around at us researchers and said, ‘well, what should we do?’

Academic It was embarrassing.

Academic And we've spent … time since the Black Report [Black, 1980] knocking down all the criticisms of what we were doing. We haven't been developing our own agenda, so I think we want to forget about being defensive and we want to say, well, these are the things which the government, if it's serious, could do.

The comments quoted above refer to the independent commission of inquiry, commissioned by the UK Labour government that was elected in 1997 into health inequalities research (Acheson 1998), which made 39 recommendations. It had, therefore, proved possible to develop some policy guidance from the available research at this time. However, these recommendations were criticised at the time for being vague and uncosted (with no hierarchy for putting the 39 recommendations in place) and for underrepresenting structural and socioeconomic determinants of health (Davey Smith *et al*. 1998). The focus group discussions drawn on here suggest that this situation has not yet improved substantially enough to enable the kind of evidence‐based policy advice that both researchers and policymakers desire (Whitehead *et al*. 2004).

### Proposals for future health inequalities research agendas

Reflecting the palpable concern with developing clearer policy guidance, when asked what researchers ought now to be focusing on, the most common responses centred on discussing means of improving knowledge about the actual and likely impacts of different kinds of policies and interventions. However, there were sharply contrasting perspectives on how best to enhance this kind of knowledge.

#### Improving evaluations

For some researchers, the need to improve knowledge about what works in reducing health inequalities necessitated a far more rigorous evaluation of interventions intended to address inequality, with a focus on their efficacy and cost‐effectiveness (see Egan *et al*. 2009, Wanless 2004). One senior academic went as far as suggesting that researchers should decline to be involved in assessing interventions or policies where a proper evaluation was not possible:

I think researchers should be tougher in saying [to policymakers], ‘if you do it like that, then we're not going to be involved in the evaluation’ … . That's my feeling, that researchers should take a stronger line, and also the politicians should, but I think researchers have a role in actually saying, ‘if you do that evaluation that way, even if you give us five million quid, we actually can't give you the answer’.

However, two linked concerns about a turn towards evaluating interventions were also evident. First, some participants claimed it was difficult to obtain resources and support to evaluate the impacts of macro‐level policy shifts or non‐health policies on health inequalities, particularly where policy changes might be expected to have a negative impact on health inequalities, such as the current welfare reforms. As a consequence, some researchers argued that a focus on evaluating interventions unintentionally leads to the widely discussed problem of ‘lifestyle drift’ (Popay *et al*. 2010):

Often the focus on … randomised control trials [and] classical intervention studies means that most of the most important determinants [are not assessed]. I mean … things to do with welfare reform and occupational structure are not going to be easily evaluated in that way.

(Academic)

Secondly, relating to this, several researchers argued that there has been a dearth of macro‐level policies implemented in the UK over the past 30 years that might be expected to reduce health inequalities and, therefore, there have been limited opportunities to evaluate policies likely to reduce health inequalities. For example:

We need to do more intervention research but part of the problem we've got, I think, over the last decade or so is that there's so few promising interventions, in truth, that have happened at a policy level that we would want to really spend a lot of time evaluating. So we've had health action zones, we've had the tobacco ban [i.e., the smoking ban in most indoor public places], we're going to have, hopefully, minimum pricing, but beyond that there's very few big policy experiments.

(Public sector researcher)

The difficulties in evaluating policy shifts and interventions in the real world, where policies are rarely implemented in ways that enable randomised comparisons, has been widely discussed in public health (Perkins *et al*. 2010). Specific suggestions from participants as to how researchers might address this included: (i) paying more attention to the impacts of policy changes and interventions on known social determinants of health, without necessarily requiring health indicators to be captured (see Bambra *et al*. 2010), (ii) working more closely with policymakers to roll out major policy changes in ways which allow for a proper evaluation and (iii) expanding methodological approaches (see below).

Despite these suggestions, for some participants it seemed that the increasing funding opportunities for evaluation orientated research were actively ‘damaging’ health inequalities research by narrowing the focus onto behavioural and individualised interventions that are easier to evaluate using positivist quantitative methodological frameworks. The concerns raised by some participants are summed up in a recent essay by Ted Schrecker:

Use of the randomized controlled trial (RCT) as the gold standard for intervention research, sitting atop a hierarchy of evidence … incorporates a set of methodological value judgments that merit reconsideration. Although examples exist of sound RCTs of large‐scale policy initiatives … many kinds of interventions and policies cannot be assessed using RCTs, for reasons of ethics, costs, logistics, or all of these. Even when an RCT is conceptually possible, insisting on evidence from RCTs may build into intervention research a bias against larger‐scale, contextual interventions that are difficult to evaluate in this manner.

(Schrecker 2013: 742)

In line with Schrecker's account, several of the more qualitative researchers involved in discussions argued that a preoccupation with evaluating interventions was squeezing out other types of research, with one senior academic referring to her sense that health inequalities research was increasingly being guarded by a ‘trial police’. For another senior academic, these concerns prompted a rejection of the very terminology associated with the evaluative turn in public health:

I've got to the point now where, for a long time I've not used the word lifestyle – it's a refusal in me to actually even use the term and I'm getting the same feeling now with the word intervention: I can't bear it when people start talking about interventions in relation to the health of deprived communities … I can't bear the imposition of the straightjacket of the kind of work we do.

The data presented in this section underline the obvious methodological tensions within the multi‐disciplinary field of health inequalities research and suggest that researchers prioritise particular methodological approaches (usually their own) over others. A likely effect of this is the compartmentalisation of research funding in ways that potentially encourage partial investigations into complex issues. The final quotation suggests there may be a related division in the kind of language that different researchers choose to employ.

#### Expanding our methodological toolkit

Most participants agreed methodological innovation, including greater use of mixed methods, is required to develop better understandings of the impacts of policy change on health inequalities but more precise suggestions differed. Some researchers were concerned that the right things are not yet being measured (for a debate on this topic, see Frank and Haw 2011, McCartney *et al*. 2013) but several (more quantitative) researchers suggested emerging data linkage opportunities could afford new opportunities for understanding the impacts of different kinds of interventions and policy changes on health inequalities:

One of the other areas which is growing in capacity and interest is the data linkage. I simply see that as … another way of being able to better evaluate social interventions, policy or whatever. Primary data collection, as we all know, is really expensive and if … we could draw on comparative stuff, qualitative stuff, primary, quantitative and … routine data across not just medical but across education, criminology, justice, then I think you could begin to get a … very rich picture of what might be working and what might not be working.

(Academic)

Overall, there seemed to be a consensus that there is a need for a more interdisciplinary approach to studying health inequalities and that researchers need to become more imaginative at incorporating different kinds of data sources into analyses (see Table 3).

**Table 3:** Researchers' suggestions for future directions in health inequalities (HIs) research

Topics requiring further research	New/emerging topics to explore	Suggested/conceptual approaches	Suggested collaborations, links & syntheses
Links between income, job status and HIs by studying the organisations in which we work (e.g., NHS bodies and universities); The impacts of social and structural violence^1^ on health inequalities; Community assets and resilience; Intervention‐generated HIs (policies and interventions that unintentionally function to widen health inequalities); How different kinds of upstream social factors lead to biological (including genetic) changes in individuals (i.e., better understanding the aetiological pathways that lead to health inequalities); Links between ‘class’ (in sociological and political terms) and HIs (see Scambler, 2012).	Likely (and actual) impacts of contemporary welfare cuts, austerity measures, etc. on health inequalities; Likely (and actual) impacts of NHS reforms (in England) on health inequalities; Develop links between environmental/climate change & HIs research; Public, political and policy perceptions and understandings of health inequalities; Role of corporations in HIs debates and in shaping the policies impacting on health inequalities; Identify more ‘intermediate’ structural determinants of HIs to enable better studies of the relationship between upstream changes and HIs (helping to guard against ‘lifestyle drift’); Investigate the relationships between violence, drugs & HIs (esp. in Scotland); Research social and economic inequalities as well as HIs (given what is known about the links between these broader kinds of inequalities and health inequalities), both because this kind of research may be more informative and because local communities are more likely to engage with it.	More mixed methods research to develop comprehensive methodological approaches to studying health inequalities; More ethnographic and anthropological methods to better understand people's life‐worlds (including using innovative approaches such as ‘human libraries’); Assess the impact of major policy shifts on HIs by employing more historical & international data; Work with policymakers to ensure commissioned evaluations are rigorous (consider refusing to undertake commissioned evaluations where this is not possible); Exploit emerging opportunities for data linkage (both in terms of quantitative data sets and linking quantitative data sets with available qualitative data); Exploit the increasing availability of secondary qualitative data sets, such as oral histories; Undertake more participatory collaborative research with relevant communities. Work to integrate studies of HIs with relevant concepts in political science, sociology and social theory.	Invest more resources in comprehensively collating/synthesising the various existing analyses of the impacts on HIs of interventions and policies. Work with policy scholars to better understand the policymaking process and the factors necessary to facilitate policy change; Work to better understand intersectionality within HIs (i.e., studying the interactions between different axes of health inequalities, such as class, ethnicity and gender); Develop more interdisciplinary/multidisciplinary research teams for HIs projects (e.g., including anthropologists, economists, education and social policy scholars, historians, sociologists and political scientists).

1 Social and structural violence refers to political and economic inequality (Farmer 1997).

NHS, National Health Service.

#### New research directions

When asked to consider new research directions, the most common theme throughout the discussions was the need for research to help understand the impacts of major policy reforms currently being implemented in the UK (e.g., public funding cuts and, in England, NHS reforms):

I think one of the things that's really important … is the absolute reality that the impact of public sector cuts and the welfare cuts which are utterly savage, I mean savage … I mean that's the reality and I think, as researchers within public health … that's a really important issue to take on board, kind of morally.

(Senior academic)

In order to better understand the impacts of economic distress and what have been labelled austerity measures on people's health and wellbeing, researchers suggested: (i) exploring historical and international experiences of similar situations (as noted above) and (ii) undertaking more in‐depth qualitative research to better understand people's lived experiences of these changes:

How good … is the research community at knowing and understanding communities and working with communities, quite genuinely listening to them? … None of us do actually really listen … . It's about actually listening to people about what it's … like for [them] where they're living and what are the challenges they face on a daily basis – which will often be different from what we think they are, because we don't know, because we're all middle class.

(Researcher)

However, another researcher challenged the above assertion, arguing instead that a particular focus on deprived communities can shift the focus away from the need for population‐level change, while also unintentionally stigmatising people living in communities labelled as deprived:

We do tend to conceptualise health inequalities as [being] about particular well‐defined communities, and of course it's all about the gradient. It's not only about the people in those communities, and sometimes I think we do harm by focusing so much on the deprived communities and calling them ‘deprived communities’ … Sometimes you make the stigma worse … because you've defined them as deprived communities and all your documents say, ‘we're focusing on people in these deprived communities’ … And I see the faces, sometimes, of the admin staff with some of the things that I write, where we say we're going to prioritise these deprived communities, and they live there! And they say, ‘but wait a minute, actually we're not deprived, we live there – it's a perfectly good community’. And I think we need to be a bit careful about saying there are some people who suffer from health inequalities and they're the deprived folk, then there's everybody else.

(Researcher)

These kinds of concerns reflect the findings of a recent seven‐country comparative study of the effects of poverty, undertaken by Walker *et al*. (2013), which argues that the shame associated with poverty is one of the most important dimensions for understanding how and why poverty impacts negatively on people's lives. Likewise, Susan George, warns researchers against studying ‘the poor and powerless’, who ‘already know what is wrong with their lives’, noting that this kind of research can be used in ways other than those intended by the sympathetic researcher (George 1976: 289).

There is, therefore, a dilemma facing health inequalities researchers; while many participants suggested it would be extremely helpful to have more in‐depth insights into the everyday experiences of living in difficult circumstances to better understand health inequalities (with several noting how different researchers' own lived experiences tend to be), there were also clear concerns around the potentially stigmatising consequences of this kind of research.

George's suggestion is that researchers ought to instead work to better understand ‘the rich and powerful’ (George 1976: 289) and several researchers supported this idea, calling for more research to understand how well‐resourced actors work to shape policies in ways that contribute to health inequalities. This is a strand of research that has so far been underexplored in health inequalities, although Scambler has drawn attention to the issue via his ‘greedy bastards hypothesis’ (Scambler 2007, 2009) which asserts that ‘Britain's widening health inequalities can be seen as a largely unintended consequence of the voracious, strategic appetites’ of capitalist power elites (Scambler 2012: 137). Specifically, several researchers argued that the role of corporations in generating health inequalities required exploration:

One thing that I think has been under‐investigated is the paradox that currently most of the immediate causes of premature mortality are due to overconsumption of, whether it's tobacco, alcohol, food and drugs, and yet the impact is the greatest on most disadvantaged groups. And it seems to me that we've not really focused enough on the free market economy and on the big multinational companies and the way in which they can exploit, particularly the weakest, to create this sense that, ‘I must have this, I need this to make me happy’, and so on, with absolutely devastating results. (Knowledge broker)

Beyond the above suggestions, which stimulated significant discussion, there was a wealth of further suggestions for topics deserving of more attention within health inequalities research. As it is not feasible to discuss each suggestion within the space of this article, Table 3 provides a summary.

It is worth noting that some researchers with interests in qualitative methods claimed that funders were increasingly more inclined to fund quantitative studies and that, ‘where qualitative aspects are included’, there was ‘a tendency for them to be seen as of marginal importance and stripped out’. This seems important given that most of the methodological suggestions put forward by researchers (as summarised in Table 3) involved qualitative or mixed methods research.

### Suggestions for improving the influence of health inequalities research on policy and practice

There was a strong emphasis throughout the discussions on the need to improve links between health inequalities research, policy and practice but clear differences of opinion regarding the best way to achieve this.

#### Getting the public on board

Reflecting recent suggestions that health inequalities researchers ought to do more to ensure future governments have the necessary democratic mandate to implement some of the upstream measures that the evidence suggests may be required (Mackenbach 2011, Whitehead and Popay 2010), many participants emphasised the need to improve relationships between health inequalities researchers and public (as opposed to policy) audiences:

We're not hugely public and most of us don't write commentaries for newspapers or letters to the papers, or do those sorts of media things that economists do all the time … There are enough of us really who have known for a long, long time that if you want to improve the public health and reduce health inequalities you've got to deal with the structural determinants, and still most members of the public don't think that. They think people die early because they're feckless, reckless, smoke and drink too much. So, we haven't got it across, not just to policymakers, we haven't got it across to the public.

(Academic)

Other researchers challenged these claims, noting that there has been insufficient research in this area to really know what the public think about health inequalities. One researcher argued that the studies of lay perceptions of health inequalities point to a relatively sophisticated understanding of the social determinants of health, at least in communities bearing the brunt of these inequalities (Popay *et al*. 2003). Nonetheless, researchers involved in the symposium seemed to feel it was at least as important to communicate and engage with local communities and the public as they did to engage with policy audiences. Indeed, some of the discussions involved some reflection as to who, ultimately, health inequalities research is for:

I am now struggling to understand who my research is for. And do I want to waste my time doing research for a government who is either going to punish me for daring to study the effects of ill‐health in housing estates, or just disregard my research? And so now I'm thinking it's kind of like an opportunity to reflect on what I'm doing, and it was a bit uncomfortable really just sitting here and listening to [symposium presentations and discussion] because they all seemed to be saying, blatantly … who is the research for?

(Academic)

The unspoken implication of these kinds of contributions seemed to be that the public, or local communities, are important agents of change, although how public and policy perceptions of health inequalities might link was not explicitly discussed.

Overall, there seemed to be a consensus that health inequalities researchers need to get better at engaging with various non‐academic communities. However, there was a distinction between researchers who emphasised the importance of listening to, and working collaboratively with, relevant communities (as the above participant went on to do) and those, such as the following researcher, who focused on the need to enhance general public interest in, and understanding of, health inequalities:

If you look back at the pioneers of social policy … Richard Titmuss, Peter Townsend, Brian Abel‐Smith … and all the rest of it, they would tell you that one op‐ed piece in a broadsheet newspaper is worth 25 peer review journal articles … Or speaking to the local radio or whatever. So I think communicating to real people in a practical way needs to be part of what's seen as our legitimate activities.

(Academic)

These two differing ways of framing engagement map onto the contrasting definitions of advocacy outlined in the introduction; while the former seems to be more about helping people to get their voices heard (facilitational advocacy – Carlisle 2000), the latter seems to be about selling particular issues and goals (representational advocacy – Carlisle 2000, Chapman 2007).

#### Getting political

A few researchers went further, arguing that it is essential for health inequalities to work more strategically to achieve social and policy change, including by developing better advocacy skills:

There's a reluctance to recognise that public health is essentially a political discipline … I do think it's indefensible that advocacy isn't a core public health competence and … I don't think it's a coincidence that lots of the pressure for advocacy being integrated into the public health curriculum has come from people like Simon Chapman [in tobacco control] who've long argued that, why is it that we're prepared to go on and make media appearance without any training, but will sit down and rehearse a conference paper that's going to be listened to by 20 people?

(Academic)

However, there was also palpable apprehension amongst some participants of the ability of academic researchers to take on advocacy roles:

I'm uncomfortable with that because … not because advocacy's wrong but because some people within the community are better suited and skilled to engage in advocacy than others. So I prefer the word grounded – I think even if you're not good at advocacy, if you're engaged in this field you should ground yourself in real everyday experiences with people in communities.

(Academic)

Some participants suggested that, rather than becoming advocates or lobbyists themselves, researchers ought simply to develop better links with other kinds of campaigners and policy advocates, such as third sector organisations. However, others expressed concern that the topic‐based nature of many third sector organisations could mean such an approach might unintentionally exacerbate ‘lifestyle drift’, and place a greater focus on ‘the ambulance at the bottom of the cliff … than your fence at the top’. These kinds of debates suggest it may also be useful to work to better understand the role of third sector organisations in health inequalities debates.

## Concluding discussion

Reflecting on the views of the 52 researchers who participated in the symposium, it seems there is a strong desire to identify clear recommendations for action to reduce health inequalities and to work collectively to promote these recommendations. Beyond this, however, three distinct types of researcher seem identifiable, each of whom has definable preferences over the most promising areas of research, the most appropriate methodological approaches and the most important non‐academic audiences to engage with. The three types are described below in the manner of Weber's (1962) ideal types (that is, the descriptions are logical constructions to help elucidate some of the coalitions and divisions that are evident in the data); in reality (as discussed further below), individual researchers often made contributions that would place them in more than one of these categories.

### Policy‐focused positivists

This type of health inequalities researcher is strongly committed to the idea that quantitative, experimental research designs provide the most useful insights into understanding what works to reduce health inequalities. In terms of moving health inequalities research forward, researchers in this category tended to promote the need for more (and better) evaluations of interventions and policies, including through the exploitation of data linkage opportunities. They came across as policy‐focused, in the sense that they want to ensure the research is useful for policy audiences (who were often conceived of in a relatively narrow sense, such as senior civil servants and ministers). However, in prioritising scientific independence and methodological rigour, it was also clear that they were wary of researchers becoming co‐opted by policymakers or interpreting data through a political or ideological lens.

This way of approaching health inequalities came under significant criticism during the symposium discussions for at least four reasons. Firstly, some researchers argued that, no matter how hard researchers might try to persuade policymakers of the benefits of rolling interventions out in ways that enable effective evaluation, major policy changes tend to be driven by reasons other than research, making it unlikely that this will change. A small number of hardline researchers in this category argued that researchers should simply refuse to engage in evaluations that they felt were likely to be hampered by the policy design or roll‐out. However, the most common, ‘softer’ response was to suggest that researchers needed to supplement traditional approaches to evaluation with broader, more sophisticated methodological approaches. Secondly, some researchers expressed concern that a greater focus on evaluation‐orientated research results, in practice, in lifestyle drift (Popay *et al*. 2010: 148), both because it is difficult to apply rigorous, quantitative evaluative methods to macro‐level (or even meso‐level) policies and because large‐scale policy shifts are relatively unusual, limiting opportunities to study the impacts of macro‐level policy changes. Thirdly, some researchers criticised the elite orientation of this way of working, which tends to focus on the views of experienced researchers and senior bureaucrats, paying little (if any) attention to the views of the communities most negatively impacted on by health inequalities (see Scambler 2012). Finally, some researchers simply noted that the benefits of this approach for reducing health inequalities remain unproven (i.e., that, despite investments in this way of working, advances in our knowledge of how to reduce health inequalities remain limited).

### Empathetic ethnographers

Other participants argued that health inequalities researchers need to interrogate what health inequalities mean in people's social worlds (Scambler 2012: 144). These researchers called for more priority to be afforded to listening to, and working to understand, the experiences of communities experiencing the brunt of health inequalities. Reflecting Ruth Lister's assertion, these participants were often overtly critical of research in which *‘*those with every day experience of living in poverty’ are treated as research objects, rather than having ‘their thoughts published’ (Lister 2004: 2). This approach was much more ethnographic in nature, with preferred methods including in‐depth qualitative methods to capture the complexity of people's everyday experiences in order to help: (i) better understand the pathways linking upstream determinants and health inequalities and (ii) prioritise research issues overtly impacting on the people bearing the brunt of health inequalities (rather than assessing what is worthy of further research based on identifying gaps in the available evidence). This group of researchers seemed committed to advocacy in the sense of working to help voices that are often ignored to become more audible (Carlisle 2000), including by co‐producing research with communities (Letcher and Perlow 2009).

In the symposium discussions, this kind of approach to studying health inequalities seemed to enjoy broad support. However, many contributors said they felt that funders were unlikely to provide resources for this kind of research. Three criticisms of this approach were also put forward, including by researchers who seemed generally sympathetic to qualitative research. Firstly, some researchers cautioned that a focus on researching poorer communities exacerbates the tendency to conceptualise health inequalities as a matter of health deprivation (i.e., a problem caused by the poor health of poor people), rather than a social gradient of health (see Graham and Kelly 2004). Secondly and relatedly, some participants argued that this can be unintentionally stigmatising for the communities involved. Thirdly, some researchers questioned whether commitments to working with communities necessarily effect change (although some successful examples were cited – see Martin *et al*. 1987, Roberts *et al*. 2011).

### Critical materialists

Taking a rather different approach, some participants called for more attention to be paid to exploring how elite actors (including corporations) shape policies and debates in ways that create and exacerbate health inequalities. For these participants, the link between health inequalities and wider social and economic inequalities seemed undisputed so their priority for future research was less about trying to understand the aetiological pathways linking policies and interventions to everyday health experiences and more about working to reveal the extent to which society is becoming less equal, who is shaping these decisions and how power relations are enacted (see Coburn 2004, Navarro 2009, Scambler 2012). It was suggested this kind of work involved drawing on social and political theory, as well as empirical research (again, see Scambler 2012), another area in which there seemed to be some consensus that funding support was limited.

From this perspective, health inequalities research is, in itself, a political activity. The notion that researchers ought to be engaging in advocacy therefore tended to be supported by researchers in this category and involved working ‘to provoke critical thinking’ and public awareness of, and agitation against, the power imbalances underlying health inequalities (see Krieger *et al*. 2012). However, it was unclear that researchers in this category had a defined sense of what they were advocating (rather than what they were against). As Scambler (2012) notes, the prospects for realising the conditions in which social and economic inequalities might be substantially reduced is a challenge more often posed than taken up. Without being able to offer obvious solutions, there are some important ethical questions to be addressed. For, if the communities in question do not have control over the levers shaping the structures that are negatively impacting on their lives, drawing attention to the health‐damaging effects of these structures may, in itself, have damaging implications (including the kind of stigmatising impacts discussed earlier).

## Where do we go from here?

The three types of health inequalities researcher outlined above are depicted as distinct from one another for heuristic purposes. In reality, it was evident from the discussions that the boundaries between these different types were not as distinct as the descriptions above might suggest; indeed, several researchers made contributions to discussions that could lead them to be positioned in more than one of these groups. Moreover, while the participants were selected with a view to ensuring a range of perspectives was represented, there are likely to be other perspectives that were not captured through this research (certainly some areas, such as health economics, were underrepresented in the sample, despite specific efforts by the steering group to attract health economists to the event). Nonetheless, the focus group discussions suggested that the three different ways of approaching health inequalities outlined above inform three different routes forward for future health inequalities research. This, in turn, appeared to be informing competing sources of policy advice (e.g., advice based on the best available evaluations of specific interventions versus advice informed by critical analyses of overarching policy paradigms) and contrasting views about with whom, beside other researchers, academics ought to be engaging (national level policymakers, local communities or the wider public).

The different approaches seem to relate to deeply held epistemological and ideological positions that are unlikely to shift. Researchers who are strongly committed to positivist approaches are unlikely, for example, to appreciate the benefits of ethnographic or co‐produced research and may dismiss critical materialist work as being overtly ideological (see Gieryn 1983, Smith 2013a). Focusing on trying to persuade each other of the benefits of our preferred way of working therefore seems ill‐fated. Yet, in the context of limited research funding, the data suggest that different types of researchers do view themselves as competing with one another. This is unsurprising, to the extent that it reflects well‐established phenomena within the sociology of science. Gieryn (1983), for example, describes scientists undertaking what he calls ‘boundary work’ in order to distinguish between what is considered to be science and what is not, with a view to enhancing the credibility of their own research and, therefore, their access to resources. This kind of boundary work was evident in the focus group discussions, with some health inequalities researchers in each of the three categories criticising methodological approaches that differed from their own and challenging the legitimacy and funding of other types of research. Such divisions seem likely to promote the continued compartmentalisation of methodological approaches to studying health inequalities, despite the fact that many researchers acknowledged there was a need for more mixed methods research to better understand the complexity of interweaving and dynamic inequalities.

All this suggests that the field of health inequalities research is unlikely to appear cohesive to external (e.g., policy and advocacy) audiences. This is despite the fact that: (i) focus group participants generally agreed that the 2008 economic crisis and subsequent 2010 austerity agenda of public cuts were likely to have important (largely negative) impacts on health inequalities; (ii) a recent online survey of researchers suggests that there are areas of consensus around policy recommendations (Smith and Kandlik Eltanani 2014) and (iii) many of the researchers involved in the symposium made contributions that would place them in more than one of these categories.

Rather than pitting these three ways of approaching health inequalities against one another a more promising approach might be to better enable space for each of these different ways of working, particularly when it comes to studying the complex effects of clusters of policy changes, such as the current austerity‐led welfare reforms. Indeed, if we consider areas of public health in which significant policy and societal changes have been achieved, such as tobacco control, there is evidence that all three kinds of approach have made important contributions (Smith 2013b). Over time, it seems plausible that different approaches may lead to similar conclusions about the most desirable (or effective) kinds of policy responses, or that a combination of insights from the various approaches may collectively contribute to reductions in health inequalities. Moreover, as Petticrew *et al*. (2004) note, different policy actors may be more or less persuaded by different kinds of evidence. This requires researchers to step back from boundary debates about what is, and what is not, considered legitimate (or even optimal) in health inequalities research. This is, however, likely to be particularly difficult in the context of an increasingly market‐based university system (Marginson and Considine 2005).

While the current situation does not make it impossible to advocate the reduction of health inequalities, it certainly makes it more challenging than recent calls imply (Mackenbach 2011), particularly as the distinct approaches to health inequalities identified in this article themselves evoke different ways of thinking about advocacy and policy change. For example (thinking back to the introduction), only empathetic ethnographers appeared to conceive of advocacy in the facilitational manner that Carlisle (2000) describes; and, while policy‐focused positivists and critical materialists both appeared to favour a representational form of advocacy, their conceptualisations of the kind of audiences and work involved in this differed in ways reminiscent of the distinction Scambler (2012) makes between policy sociology and critical sociology. Hence, without (at the very least) a greater appreciation of these different ways of working in the research community, it is likely to be difficult for researchers to contribute to developing effective advocacy for health equity (Farrer *et al*. 2015).
